# Modulation of ZnO Nanostructure for Efficient Photocatalytic Performance

**DOI:** 10.1186/s11671-022-03760-x

**Published:** 2022-12-09

**Authors:** Peng Long, Hao Peng, Bolin Sun, Jinshen Lan, Jing Wan, Yuchen Fei, Xiaofang Ye, Shanzhi Qu, Gengnan Ye, Yilin He, Shengli Huang, Shuping Li, Junyong Kang

**Affiliations:** Engineering Research Center of Micro-Nano Optoelectronic Materials and Devices, Ministry of Education, Fujian Key Laboratory of Semiconductor Materials and Applications, CI Center for OSED, Department of Physics, Jiujiang Research Institute, Xiamen University, Xiamen, 361005 China

**Keywords:** ZnO, Nanostructure, Photocurrent response, Photocatalysis

## Abstract

Structure has been considered to play an important role in photocatalytic performance of the semiconductors, but the intrinsic factors were rarely revealed. Herein, ZnO nanomaterials in the structures of thin film, nanowire array and nanosheet array were synthesized, and their structural characteristics, optical properties, photocurrent response and photocatalytic efficiency were compared with each other for illustrating the issue. The photoluminescence intensity decreased in the order of nanosheets, thin film and nanowires for improved lifetime of the photoexcited charges. The absorption of the nanosheets and nanowires improved obviously in the visible range with a redshift of the absorption edge than that of the thin film. The nanowires possessed the highest response current of 82.65 μA at a response time of 2.0 ms in a sensitivity of 87.93 at the light frequency of 1 Hz, and gained the largest catalytic efficiency of 2.45 μg/cm^2^ h for the methylene blue degradation in UV light. Nevertheless, the improvement of catalytic efficiency of the nanosheets (up to 42.4%) was much larger than that of nanowires (5.7%) and thin film (2.6%) for the Au coating. The analysis revealed that the photocatalytic efficiency of the ZnO nanomaterials was modulated by the structure as it contained different surface area, roughness, defect and doping states, vacancies, polar and non-polar crystalline faces, which would provide structural design of semiconductor nanomaterials for the photoelectric and photocatalytic applications.

**Supplementary Information:**

The online version contains supplementary material available at 10.1186/s11671-022-03760-x.

## Introduction

With the development of nanotechnology, the design and regulation of nanoscale materials have been in a great progress, which are currently applied in various fields like energy, catalysis, medicine, sensing, etc. Compared with bulk materials, nanomaterials possess many superior properties and functionalities due to scale reduction. For example, the bandgap of a semiconductor would be widened by the decreasing dimension [1, 2]. The electrons in the metal nanoparticles (NPs) would be expelled to the surface as the localized surface plasmon by the light irradiation [3, 4]. It is well-known that semiconductor nanomaterials can be catalysts for the dye photodegradation. The metal NPs coating and metal doping can improve the photocatalytic efficiency significantly [5–12]. Besides the limitation of redox potential higher than that of the dye molecules [13], the structure of the semiconductors plays an important role in the photocatalytic performance [14–16]. The large surface area of the semiconductor nanomaterials is usually considered to dominate the performance [17–19], but the other intrinsic factors were rarely revealed.

With above consideration, in this study ZnO nanomaterials in the structure of thin film, nanowire array, and nanosheet array were fabricated by using different methods. The physicochemical properties of the different ZnO nanostructures were compared with each other, in addition to the applications in photodetection and photocatalysis, as well as the catalytic stability. The effect of metal NPs on photocatalytic activity of the specimens was also investigated systematically.

## Experimental

### Materials

Indium tin oxide (ITO, In:SnO_2_, 7–10 Ω, 20 × 20 mm^2^ active area) glass substrates were ordered from Luoyang Guluo Glass Co. Ltd (Henan, China). Zinc nitrate hexahydrate (Zn(NO_3_)_2_·6H_2_O, 99.5%), potassium chloride (KCl, 99%), methylene blue (MB, 98.5%), zinc acetate dihydrate (Zn(CH_3_COO)_2_·2H_2_O, 99%), and hexamethylenetetramin (C_6_H_12_N_4_, 99.5%) were all ordered from Xilong Chemical Co. Ltd (Guangdong, China). Diethyl zinc (Zn(C_2_H_5_)_2_, 99.999%) for atomic-layer deposition (ALD) was bought from Ke-Micro Company (Jiaxing, China). Deionized water (H_2_O) with a resistivity higher than 18.0 MΩ cm was supplied by a Hi-tech laboratory water purification system. All chemicals and solvents used were at least reagent grade without any additional purification.

### Thin Films

The ZnO thin films were grown on ITO substrates by a TALD-100-2H1R ALD system from Ke-Micro Company (Jiaxing, China). The substrates were cleaned in turn by deionized water, absolute ethanol, and acetone to remove inorganic and organic impurities that might exist on the surface. The Zn(C_2_H_5_)_2_ and H_2_O were used as Zn and oxygen sources, respectively. In a specific process, the ITO substrates were placed in the chamber, in which the temperature and pressure were maintained at 150 ℃ and 0.15 torr. Afterward, 200 circles of Zn(C_2_H_5_)_2_ dose for 0.02 s, N_2_ flow for 25 s, water vapor dose for 0.015 s, and N_2_ flow for 25 s were applied to grow the thin film in a thickness of 30 nm. The substrates were finally annealed at 500 °C in air. For comparison, some substrates with the thin film were deposited by a layer of Au NPs in a thickness of 10 nm by a SCD005 ion sputter coater.

### Nanowires

The ZnO nanowire arrays were synthesized by a simple chemical bath deposition method with the thin films on the ITO substrates as the seed layer [17]. The substrates were placed downward in a mixed aqueous solution of 0.02 M Zn(CH_3_COO)_2_ and 0.02 M C_6_H_12_N_4_ at 95 °C for 2 h. Then, the substrates were taken out and washed with water to remove excess salt, and annealed at 500 °C in air. The active area of the nanowire substrate was only 20 × 15 mm^2^. For comparison, some substrates with the nanowires were deposited by a layer of Au NPs in a thickness of 10 nm by the sputtering.

### Nanosheets

The ZnO nanosheet arrays were prepared by a common electrochemical deposition method [20, 21]. The ITO substrates were firstly cleaned ultrasonically in an aqueous solution of 9.7% NaOH for 30 min, then cleaned by deionized water and dried in air. Secondly, the ZnO sheets were deposited on the ITO substrate by the electrochemical deposition method, in which the ITO substrate as the working electrode, Pt plate as the counter electrode, saturated calomel electrode as the reference electrode, and a mixed aqueous solution of 0.05 M Zn(NO_3_)_2_ and 0.1 M KCl as the deposition solution. In a working potential of − 2.0 V, the ZnO nanosheets grew on the substrate at 50 °C for 40 min. Thereafter, the excess salt was rinsed off from the substrates with slow-flowing water. Finally, the substrates were annealed at 350 °C in air. The active area of the nanosheet substrate was 20 × 16 mm^2^. For comparison, some substrates with the nanosheets were deposited by a layer of Au NPs in a thickness of 10 nm by the sputtering.

### Characterization

Surface morphology, crystal structure and elemental components of the specimens were characterized by a ZEISS Sigma scanning electron microscope (SEM), an energy dispersive spectroscope (EDS) equipped in the SEM, and a X’Pert PRO X-ray diffractometer (XRD) with Cu Ka radiation (*λ* = 1.54056 Å); photoluminescence (PL) spectra were collected on a Hitachi F-7000 fluorescence spectrophotometer with an excitation wavelength of 325 nm. Absorption spectra were tested on an Agilent Carry-5000 UV–Vis–NIR spectrophotometer. Photocurrent response was measured in a three-electrode electrochemical cell by an electrochemical workstation (Chenhua CHI660E, China), with KCl solution (1 mol/L, 100 mL) as electrolyte, the specimens as a working electrode, a platinum plate (area: 1 cm^2^) and a Ag/AgCl wire as a counter electrode and a reference electrode, and an ultraviolet light-emitting diode (UV-LED) in a wavelength of 267 nm as the light source, respectively. The bias voltage of the working electrode was 0.1 V, and the given potential of the LED was 7.5 V in a square wave with the frequencies of 1, 10, and 100 Hz. Photocatalytic activity of the samples was evaluated by the degradation of MB dye under the light irradiation of a 500 W high-pressure mercury lamp (at the dominant emission wavelengths of 365 nm and *λ* ≥ 380 nm, respectively) at ambient temperature. For the measurement, aqueous suspension of MB solution (1 × 10^−5^ M) in a volume of 5 mL was poured into a glass Petri dish with a diameter of 32 mm, then a piece of sample was immersed in the solution. They were subjected to UV light and simulated sunlight in an intensity of about 1050 μW/cm^2^ and 500 μW/cm^2^, respectively. The photodegradation was performed 1 h per time, and three successive reaction periods on the MB solution with identical concentration were conducted for each sample. The photocatalytic efficiency was analyzed by measuring absorption spectra of the degraded MB solutions by the Carry-5000 spectrophotometer.

## Results and Discussion

### Structure Modulation

**Fig. 1:**
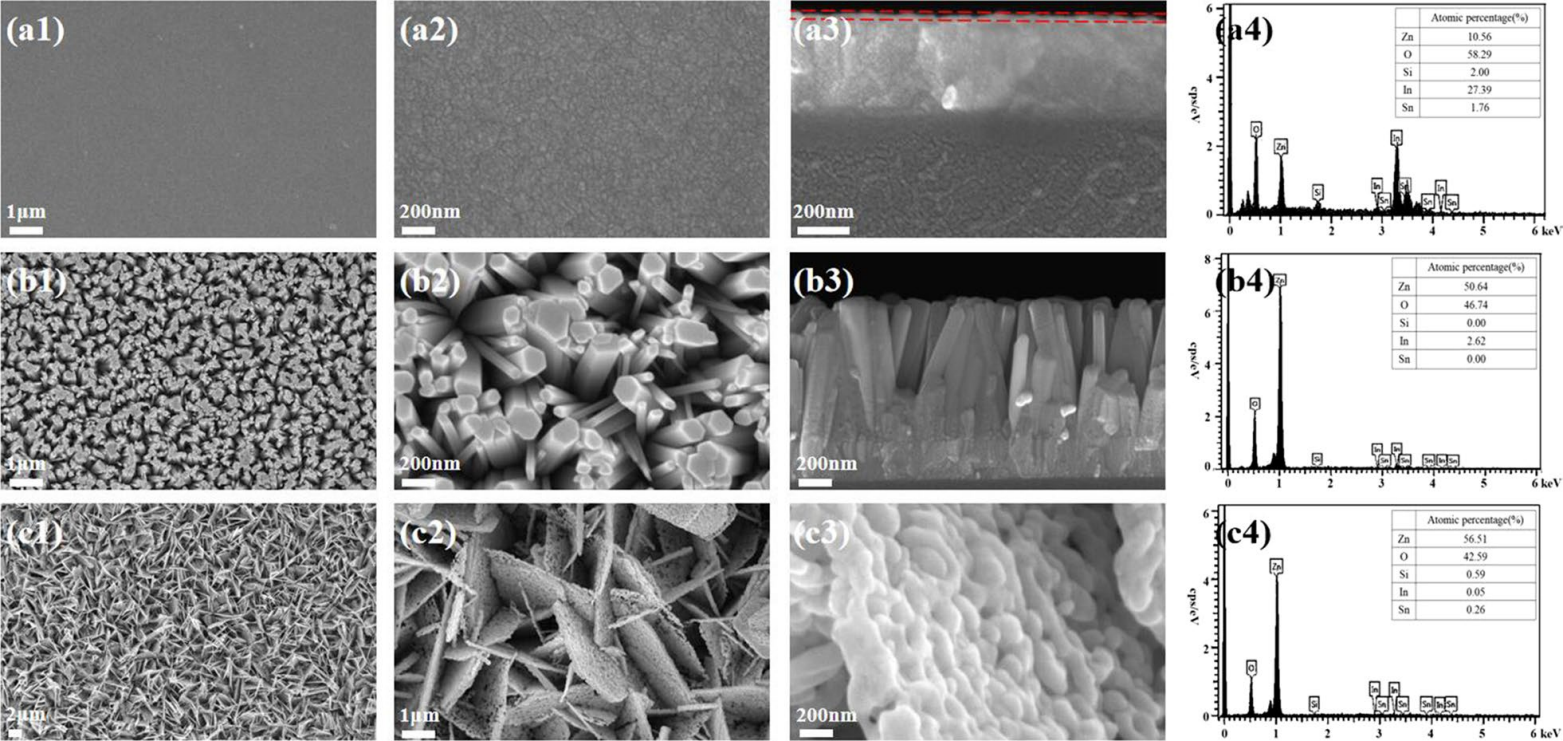
SEM images and EDS spectra of the specimens: **a1**–**a4** thin film, **b1**–**b4** nanowires, **c1**–**c4** nanosheets. The dot lines in **a3** point out the area of ZnO film

Structure images and element components of the different ZnO nanostructures are shown in Fig. 1. The ZnO thin film in Fig. 1a1, a2 is composed of dense NPs with a size of about 5–25 nm. The NP morphology is irregular, as those deposited by the sol–gel spin-coating process [22]. The side-view image in Fig. 1a3 reveals a three-layer structure that includes a glass layer in the bottom, an ITO (In:SnO_2_, about 400 nm) coating film in the middle, and a thin film of ZnO on the top. The thickness of the ZnO film is about 30 nm. The small amount is also verified by the EDS spectrum in Fig. 1a4, in which a low proportion of Zn atoms is observed with a high proportion of In and O atoms. The ZnO nanowires in Fig. 1b1–b3 nearly grow normal to the substrate. They have a length of about 600 nm with different diameters, mainly in the range of 50–150 nm as well as a small amount in 17–20 nm, The atomic percentage of Zn is significantly improved in Fig. 1b4, indicating that the ZnO nanowires are long enough to shield the substrate and contain lots of oxygen vacancies in the crystal. The ZnO nanosheets in Fig. 1c1, c2 are primarily in a hexagonal structure with a diagonal of 1.5–3.0 μm and a thickness of 50–100 nm. They stand vertically and densely with sheet edge on the substrate. In a large magnification in Fig. 1c3, the nanosheets are found to be composed of NPs in a melting state. The ratio of Zn:O is around 1.33, bigger than that of ZnO nanowires, suggesting the presence of oxygen vacancies with a larger amount in the nanosheets.

**Fig. 2:**
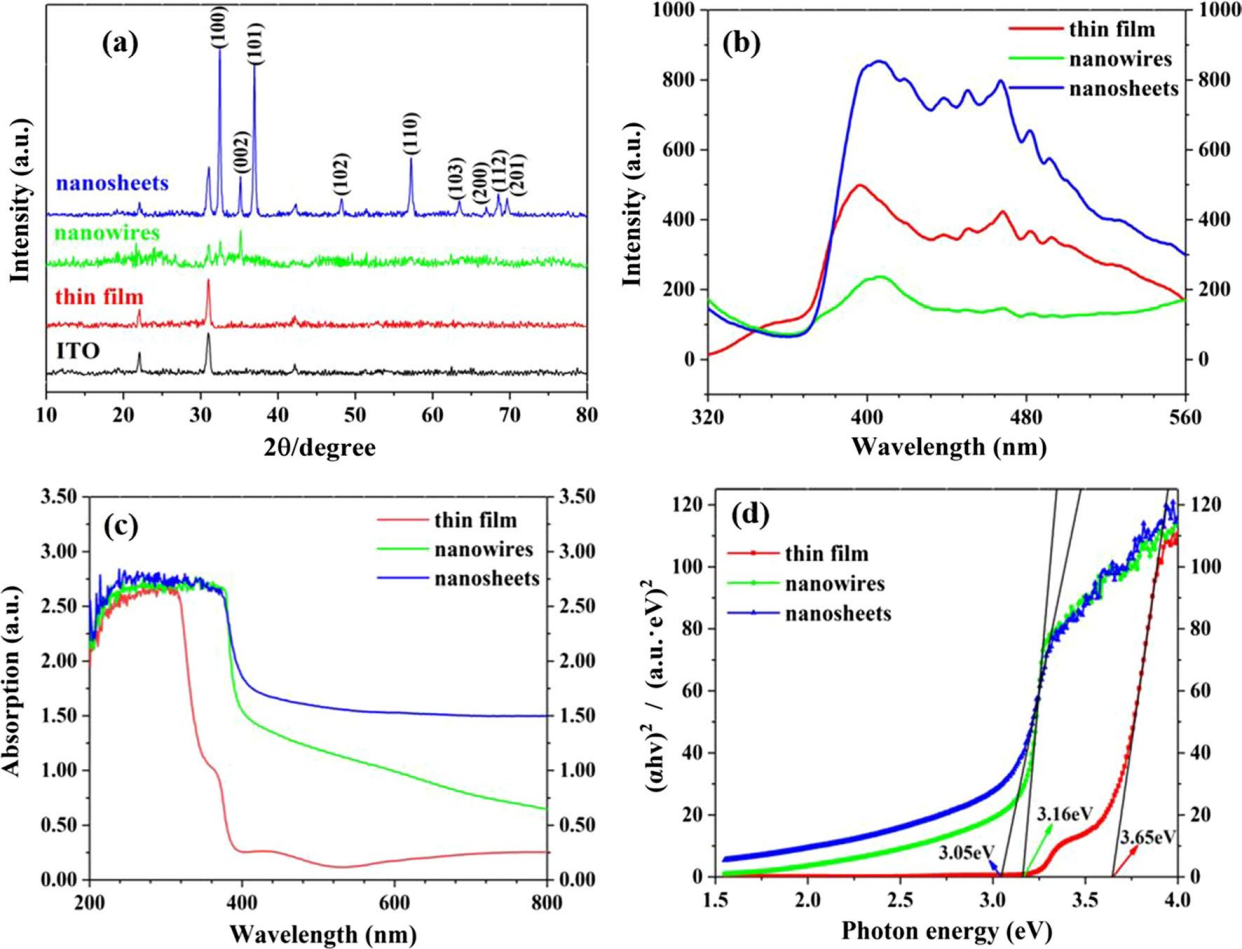
Optical spectra of the specimens: **a** XRD, **b** PL, **c** absorption, **d** Tauc plots

XRD spectra of the ZnO nanostructures are presented in Fig. 2a. Except the characteristic peaks of the ITO substrate, all the remaining diffraction peaks of the ZnO nanosheets can be assigned to hexagonal wurtzite structure (JCPDS NO. 36-1451). The strongest peaks at the 2θ values of 31.8° and 36.3° correspond to the (100) and (101) crystal planes, respectively, indicating that the nanosheets mainly grow along these two directions. There are other relatively weak peaks for the different crystal plane orientations. All these are in good agreement with SEM images of the sample in Fig. 1c2, c3, where overlapping and mixed growth orientations of the crystal planes can be seen. For the ZnO nanowires, only the peaks of (100) and (002) plans are observed, and their peak intensity is very weak, suggesting that the crystal quality of the ZnO nanowires is not as good as that of nanosheets. Moreover, the (100) and (110) planes correspond to the non-polar plane (11$\overline{2}$0), while the (002) plane corresponds to the polar plane (0001), and the polar plane is prone to the creation of Zn vacancies and O vacancies [23]. The relatively strong intensity of the polar plane in the nanowires illustrates facile creation of the vacancies. Nevertheless, no characteristic peaks of the ZnO crystal are observed for the thin film, which may result from the limited thickness or the amorphous state of the specimen.

### Optical Properties

PL spectra of the specimens are shown in Fig. 2b. All the ZnO nanomaterials display a strong luminescence peak at 375–410 nm and a series of blue luminescence peaks in the range of 420–500 nm. The luminescence peak in the UV region is related to the direct recombination of photogenerated carriers between the conduction band and valence band [24–26], corresponding to the intrinsic emission of ZnO nanomaterials. While the blue luminescence peaks can be attributed to the recombination of the electrons in shallow donor energy levels and holes in valence band, which originates from the n-type semiconducting properties of ZnO itself, as Zn interstitials or Zn inversions lead to the n-type properties of undoped ZnO and the lower formation energy of O vacancies [27]. The intensity of the intrinsic emission and blue emissions decreases in the order of nanosheets, nanofilm and nanowires. The lowest intensity of the intrinsic emission of the nanowires suggests the longest lifetime of the photoexcited charges, which is advantageous for the photoelectric and photocatalytic performance. The coating of Au NPs on the ZnO nanostructures brings depression of the fluorescence intensity, as clearly observed in Additional file 1: Fig. S1. The fluorescence intensity of the Au/nanosheets reduces the most and gets close to that of the Au/thin film followed by the Au/nanowires. The reduced intensity indicates extending lifetime of the photoexcited charges, which may be due to the Schottky junction between the Au and ZnO that separates the electrons and holes effectively by the built-in potential.

Figure 2c presents absorption spectra of the samples. The ZnO thin film owns a strong absorption in the UV range and a low absorption in the visible range, in addition to an absorption edge at about 370 nm. Compared with the thin film, the absorption edge of the nanosheets and nanowires takes a redshift significantly, and the absorption intensity is obviously enhanced in the visible range. The Au NPs increases the absorption intensity in the visible range, while the increase is barely visible in the UV range (see Additional file 1: Fig. S2). The bandgap (*E*_g_) of the ZnO nanostructures can be estimated by the Tauc plot [28],

1 $$\left( {\alpha h\nu } \right)^{2} = A\left( {h\nu - E_{{\text{g}}} } \right),$$

where *α* is the absorptivity, $h\nu$ represents the incident photon energy, and *A* is the correlation constant. As shown in Fig. 2d, the achieved bandgaps of thin film, nanowires and nanosheets are 3.65, 3.16 and 3.05 eV, respectively. In contrast to bulk ZnO (3.37 eV) [29], the bandgap of the thin film is broadened, while that of nanowires and nanosheets is narrowed down, especially for the nanosheets. The wide bandgap of the thin film may result from the space restriction of the ITO substrate and the limited film thickness, as the small size can lead to bandgap widening of the semiconductors [1, 2]. Nevertheless, the nanowires and nanosheets grow normal to the substrate and in the free space. The bandgap of both the samples may be reduced in contrast to that of bulk ZnO for the ion doping and vacancy in the chemical bath deposition and electrochemical deposition, though the impurities are undetectable in the EDS spectra in Fig. 1b4, c4 for the low content. The smaller bandgap means that the nanowires and nanosheets would have better utilization efficiency of the visible light.

**Fig. 3:**
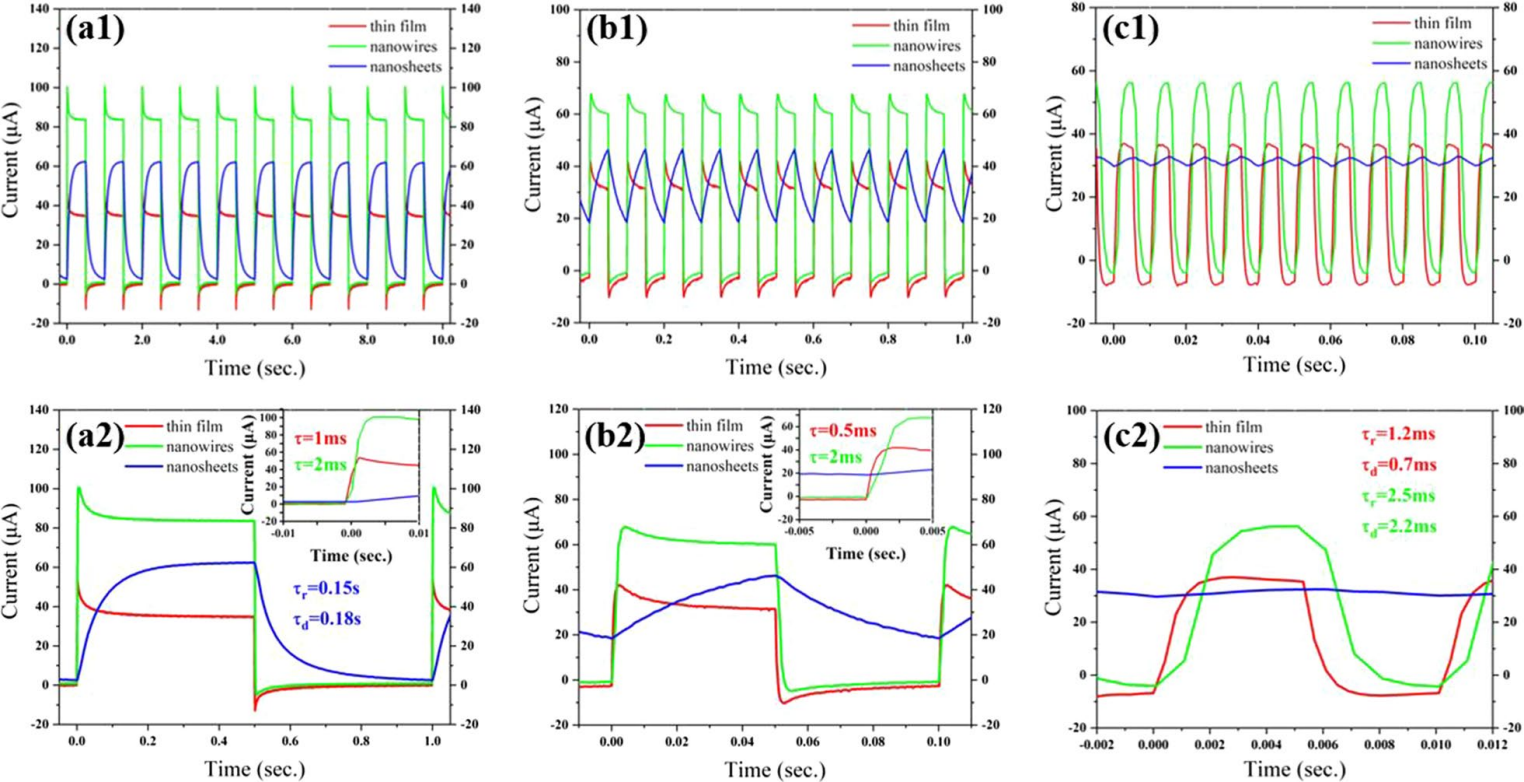
Photocurrent response of the specimens under UV light irradiation in a square wave with different frequency: **a1**, **a2** 1 Hz, **b1**, **b2** 10 Hz, **c1**, **c2** 100 Hz

Figure 3 displays photocurrent response of the specimens, in which the response parameters are supplied in Table 1. Under UV light irradiation, the photocurrent of the nanomaterials increases immediately, especially for the thin film and nanowires (tens of μA). In contrast to the nanosheets, the thin film and nanowires possess stronger photocurrent with faster recombination. The current intensity and response time are also modulated by the light frequency. For the thin film, the response current (the difference between the stable maximum value, *I*_max_ and the stable minimum value, *I*_min_) is 34.79 μA, and the response time is as low as ~ 1.0 ms (starting from the stable minimum current to the current value of 90% maximum) at the frequency of 1 Hz, which gains a sensitivity as high as 579.83. With increasing frequency, the response current and response time of the specimen decrease a lot at 10 Hz but amplify obviously at 100 Hz, while the sensitivity reduces continuously. Moreover, the rising time (*τ*_r_) and declining time (*τ*_d_) of the photocurrent keep the same at 1 and 10 Hz but become different with *τ*_r_ > *τ*_d_ at 100 Hz. For the nanowires, the *I*_max_, response current and response time are 83.59 μA, 82.65 μA, 2.0 ms, respectively, in a sensitivity of 87.93 at the frequency of 1 Hz, which is more sensitive and faster than many reported photodetectors based on ZnO nanostructures [30, 31]. The current intensity and sensitivity of the specimen reduce monotonously with increasing frequency, which may be caused by the reduction in photogenerated carriers in each cycle. At 100 Hz the *τ*_r_ and *τ*_d_ of the photocurrent are also different with *τ*_r_ > *τ*_d_. For the nanosheets, the photocurrent is much poorer than that of others as the response time is much longer than 100 ms at 1 Hz, though the *I*_max_ and response current are larger than those of nanofilm. The *I*_max_, current intensity and sensitivity reduce significantly and continuously with increasing frequency. The *τ*_r_ and *τ*_d_ of the photocurrent become difference with *τ*_r_ < *τ*_d_ at 1 Hz and turn larger than the half period of the square light. The declining photocurrent response may be ascribed to the relatively poor carrier generation and fast recombination of the nanosheets for the extraordinary arrangement of the NPs in Fig. 1c2.

**Table 1:** Photocurrent response of the specimens under UV light irradiation in a square wave with different frequency at a constant bias voltage of 0.1 V

Structures	Light frequency (Hz)	*I*_min_ (μA)	*I*_max_ (μA)	Response current (*I*_max_ − *I*_min_, μA)	Response time (*τ*_r_/*τ*_d_)	S*
Thin film	1	0.06	34.85	34.79	1.0/1.0 ms	579.83
	10	− 2.57	31.43	34.00	0.5/0.5 ms	13.23
	100	− 6.78	35.28	42.06	1.2/0.7 ms	6.20
Nanowires	1	0.95	83.59	82.65	2.0/2.0 ms	87.93
	10	− 0.86	60.03	60.89	2.0/2.0 ms	70.80
	100	− 4.06	56.25	60.31	2.5/2.2 ms	14.85
Nanosheets	1	2.57	62.41	59.84	0.15/0.18 s	23.28
	10	18.38	46.22	27.84	–	1.51
	100	29.69	32.46	2.77	–	0.09

*The sensitivity is calculated according to the equation, $S = \left| {\frac{{I_{\max } - I_{\min } }}{{I_{\min } }}} \right|$

### Photocatalytic Performance

**Fig. 4:**
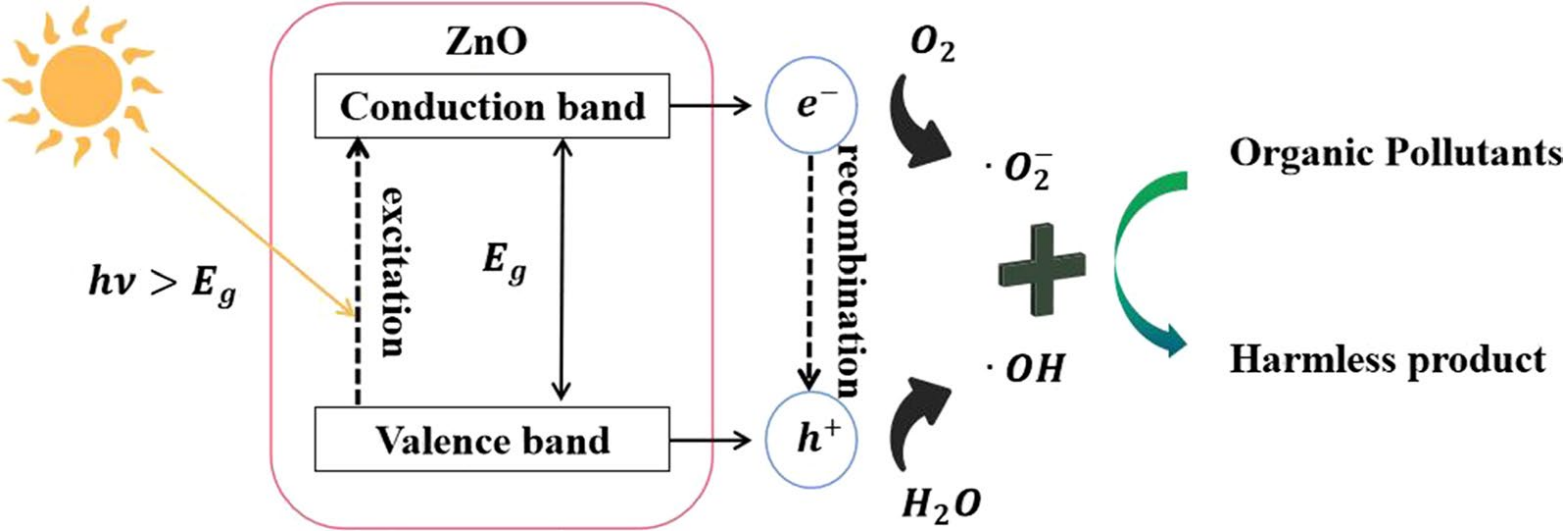
Schematic diagram of the photocatalytic process of ZnO nanomaterials

A common photodegradation process may occur in the following four steps: (1) absorption of photons to generate charge carriers (electron–hole pairs); (2) transferring of the separated charges to the catalyst surface; (3) creation of the active radicals at the surface; (4) redox reactions between the radicals and the pollutant molecules at the surface [32]. This means that for a good catalyst, its charge separation ability and surface charge assembly are particularly important. For the ZnO nanomaterials, the general process of the photocatalysis is shown in Fig. 4. That is, ZnO nanomaterials absorb photons with energy larger than its band gap (*E*_g_) to generate charge carriers. Thereafter, the charges transfer to the catalyst surface. On the surface, the electrons (**e**^**−**^) and holes (**h**^**+**^) react with O_2_ and H_2_O to generate active radicals, such as hydroxyl groups (·OH) and superoxide ($\cdot {\text{O}}_{2}^{ - }$). Finally, the pollutants are decomposed into harmless substances by the redox reaction of active radicals and organic molecules.

**Fig. 5:**
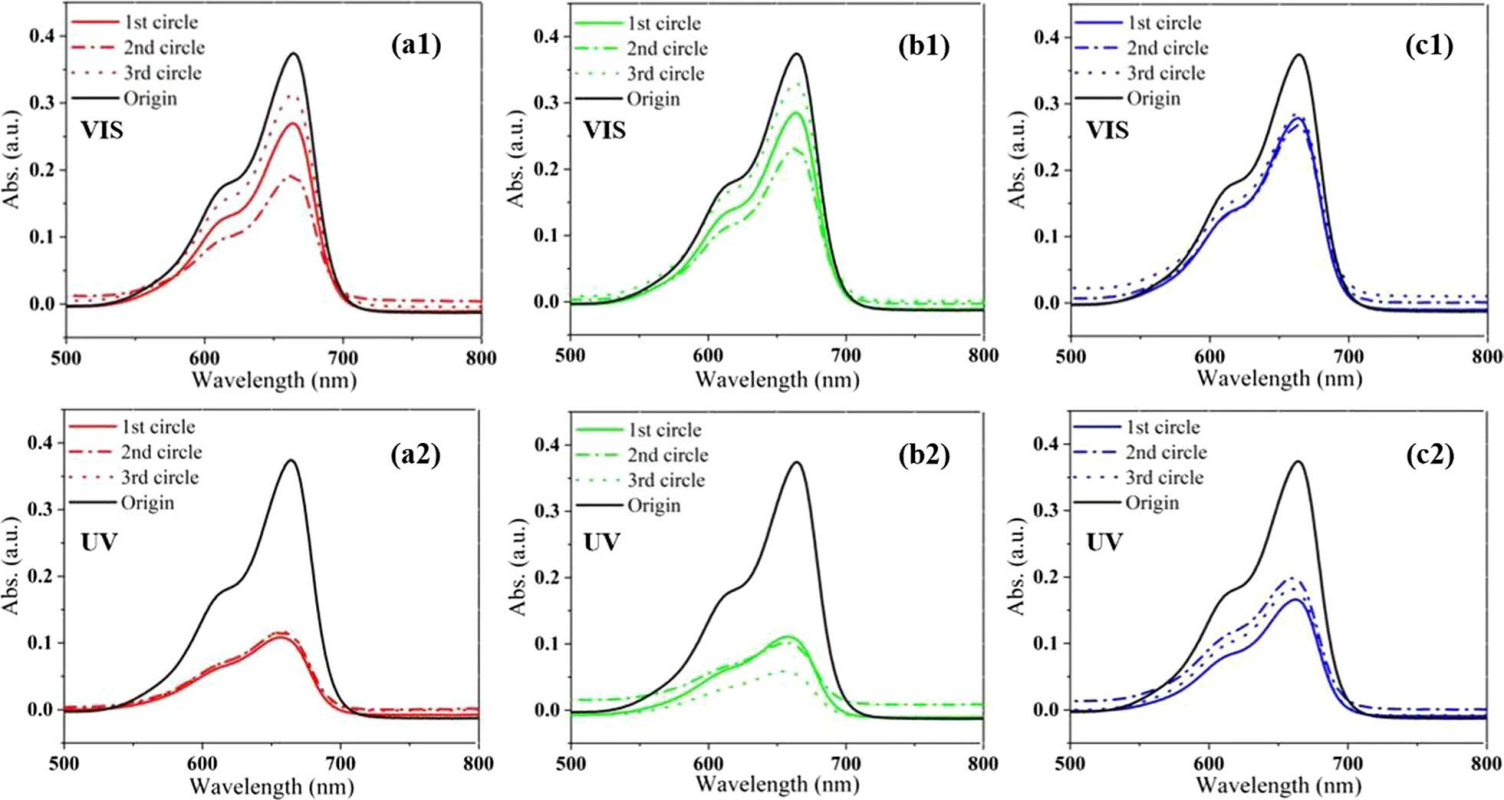
Absorption spectrum of the degraded MB solution as catalyzed by the specimens under visible light (top row) and UV light (bottom row) in 3 cycles: **a1**, **a2** thin film, **b1**, **b2** nanowires, **c1**, **c2** nanosheets

Figure 5 shows absorption spectra of the degraded MB solutions as catalyzed by the ZnO nanostructures. In comparison with the original solution, the spectral intensity of the degraded solution is reduced obviously, no matter in visible light or in UV light, indicating photocatalytic ability of the specimens. The photocatalytic performance of the specimens in the visible light may be ascribed to the carbon doping, oxygen vacancies and defect states in the materials, which create some localized energy states in the forbidden bandgap and bring charge excitation from the states to the conduction band, as observed in lots of metal oxides [25, 33], while surface O vacancies have been shown to act as photogenerated electron trapping centers to enhance electron–hole separation for enhanced photocatalytic activity [34]. Under visible light the spectral intensity of the solution as catalyzed by the thin film and nanowires fluctuates significantly in three cycles (1 h of each circle), while that of the nanosheets almost remains the same. However, the spectral intensity turns to be more stable in the UV light for all the samples, and becomes much weaker than that of the degraded solution in the visible light. This suggests improved catalytic stability and efficiency of the ZnO nanostructures under the UV light condition.

**Fig. 6:**
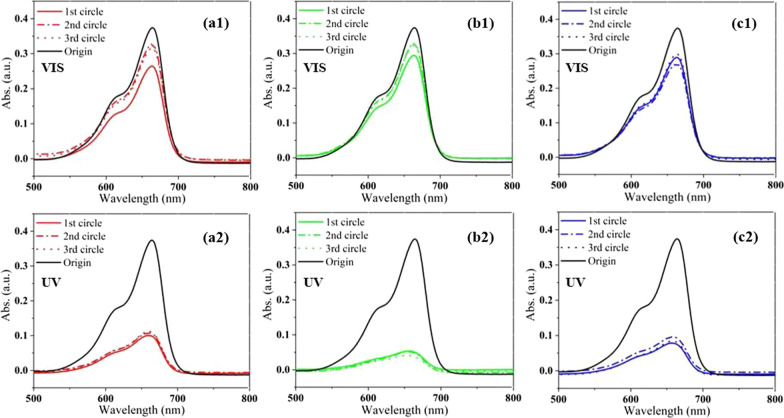
Absorption spectrum of the degraded MB solution as catalyzed by the Au-specimens under visible light (top row) and UV light (bottom row) in 3 cycles: **a1**, **a2** Au/thin film, **b1**, **b2** Au/nanowires, **c1**, **c2** Au/nanosheets

Many studies have shown that the modification of semiconductors by metal NPs is significant to enhance the photocatalytic performance. Au NPs attached to the surface of ZnO have been reported to produce surface plasmon resonance effect and increase the photocatalytic efficiency of ZnO [7, 8], that is, promoting charge generation and prolonging electron–hole pair separation in the light irradiation, thereby improving the light utilization and catalytic performance of the ZnO. Figure 6 displays absorption spectra of the degraded solution as catalyzed by the ZnO nanomaterials covered with Au NPs. The modification of ZnO by the Au NPs really leads to a downward intensity (an increasing catalytic efficiency) in the UV light region, but an upward intensity (a decreasing catalytic efficiency) in the visible light region. The declining efficiency in the visible region may be that the Au NPs is excessive, which shields the ZnO nanomaterials and reduces utilization of the visible light, though the Au NPs enhance the absorption of visible light (see Additional file 1: Fig. S2). Nevertheless, the vibration of the spectral intensity becomes smaller in the three cycles for all the samples than those without Au NPs in Fig. 5, suggesting improvement of the catalytic and structural stability of the specimens for the Au coating.

**Fig. 7:**
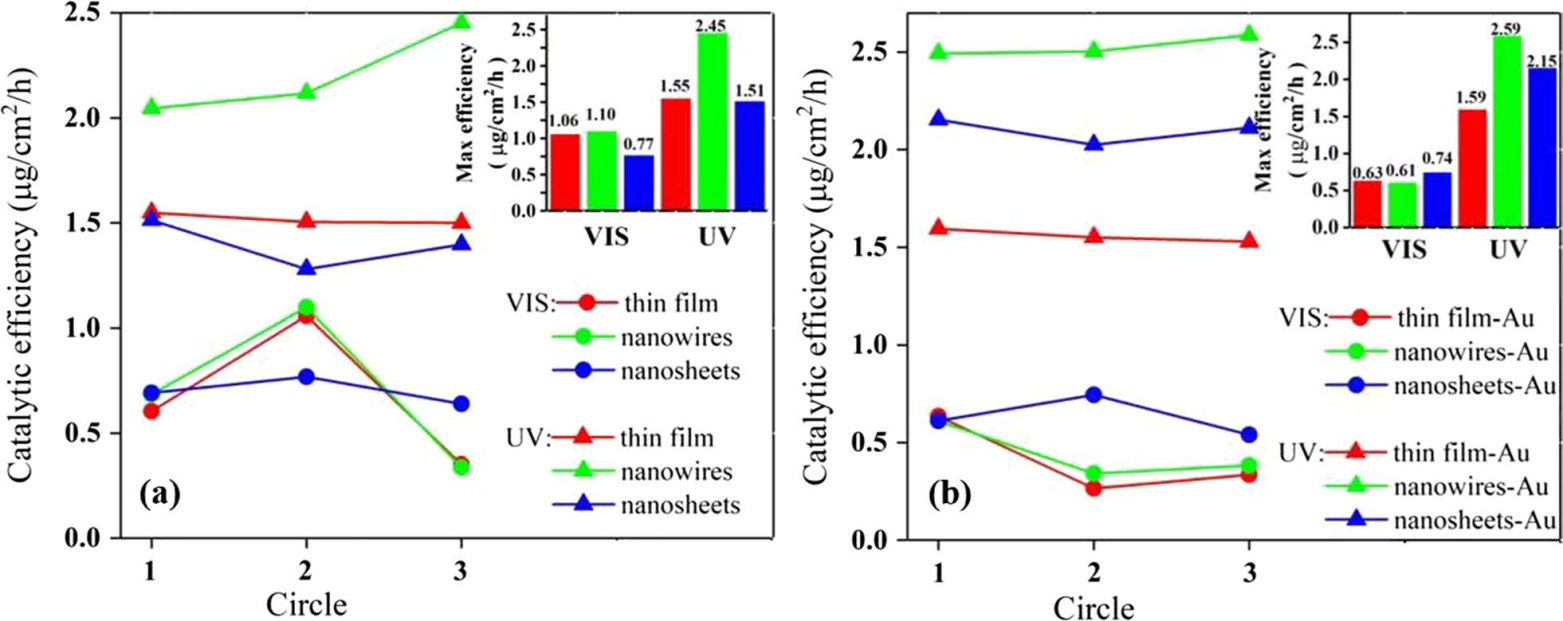
Evolution of photocatalytic efficiency in 3 cycles under visible light and UV light for the specimens: **a** ZnO without gold NPs, **b** ZnO with gold NPs. The inset is the maximum efficiency in the three cycles

The catalytic efficiency is calculated by considering the band intensity of the solution and the active area of the substrate surface, and is shown in Fig. 7. The efficiency in the UV light is much larger than that in the visible light for all the samples with and without Au NPs, which is because more charge carriers are generated by the higher photon energy of the UV light and join the catalytic performance. For the samples without Au NPs, the ZnO nanowires possess the largest efficiency of 2.45 μg/cm^2^ h and an upward trend with the cycle process. The ZnO nanosheets present the poorest photocatalytic performance in the UV and visible light, probably because they are mainly grown along the non-polar faces, which is different from previous studies where ZnO tends to grow along polar planes [23]. For the samples with Au NPs, the ZnO/Au nanowires also own the largest efficiency of 2.59 μg/cm^2^ h in the UV light. However, the catalytic efficiency of ZnO nanosheets improves significantly from 1.51 μg/cm^2^ h without Au NPs to 2.15 μg/cm^2^ h with Au NPs in the UV light. The change (up to 42.4%) is much bigger than that of nanowires (5.7%) and thin film (2.6%) in the UV light, which should be due to the larger and rougher surface and the more extension of carrier lifetime of the nanosheets than the other two samples (see Additional file 1: Fig. S1) for the deposition of Au NPs. With above analysis, it is found that the photocatalytic efficiency of ZnO nanomaterials could be effectively modulated by the structure as it is closely related to the surface area, roughness, defect and doping states, vacancies, polar and non-polar crystalline faces. The ZnO nanostructure with large surface area, a moderate surface roughness, suitable defect and doping states as well as surface oxygen vacancies, crystallization in polar face, and appropriate metal NPs modification would own an excellent photocatalytic performance for the organic degradation.

## Conclusion

ZnO nanomaterials in the structures of thin film, nanowire and nanosheet were successfully synthesized on the ITO glass substrates. All the ZnO nanomaterials displayed a strong luminescence peak and a series of blue luminescence peaks, but the peak intensity decreased in the order of nanosheets, nanofilm and nanowires for the improved lifetime of the photoexcited charges. The nanowires possessed the highest response current of 82.65 μA at a response time of 2.0 ms in a sensitivity of 87.93 at the frequency of 1 Hz, which was more sensitive and faster than many reported photodetectors based on ZnO nanostructures. The ZnO nanowires possessed the largest catalytic efficiency for the MB degradation, but the efficiency change of ZnO nanosheets was much larger than that of others for the Au NPs coating in the UV light. The photocatalytic efficiency of the ZnO nanomaterials was found to be modulated by the structure as it contained different surface area, roughness, defect and doping states, vacancies, polar and non-polar faces, which shed light on the design of semiconductor nanomaterials for the photoelectric and photocatalytic applications.

## Supplementary Information

**Additional file 1.** PL and absorption spectra of the ZnO nanostructures with and without Au NPs.

## Data Availability

All data supporting the conclusions of this article are included within the article.
