# Supplementary material for: Modulation of ZnO Nanostructure for Efficient Photocatalytic Performance
Source: Nanoscale Res Lett. 2022 Dec 9;17:118. doi: 10.1186/s11671-022-03760-x (PMC9733757; doi:10.1186/s11671-022-03760-x)
Supplement: Supplementary file 1 — Additional file 1. PL and absorption spectra of the ZnO nanostructures with and without Au NPs. [file 11671_2022_3760_MOESM1_ESM.pdf]

Modulation of ZnO nanostructure for efficient photocatalytic performance

Peng Long, Hao Peng, Bolin Sun, Jinshen Lan, Jing Wan, Yuchen Fei, Xiaofang Ye, Shanzhi Qu, Gengnan Ye, Yilin He, Shengli Huang,* Shuping Li,* Junyong Kang

Engineering Research Center of Micro-nano Optoelectronic Materials and Devices, Ministry of Education, Fujian Key Laboratory of Semiconductor Materials and Applications, CI Center for OSED, Department of Physics, Jiujiang Research Institute, Xiamen University, Xiamen 361005, China

*Corresponding authors, E-mail: huangsl@xmu.edu.cn (S. Huang) and lsp@xmu.edu.cn (S. Li).

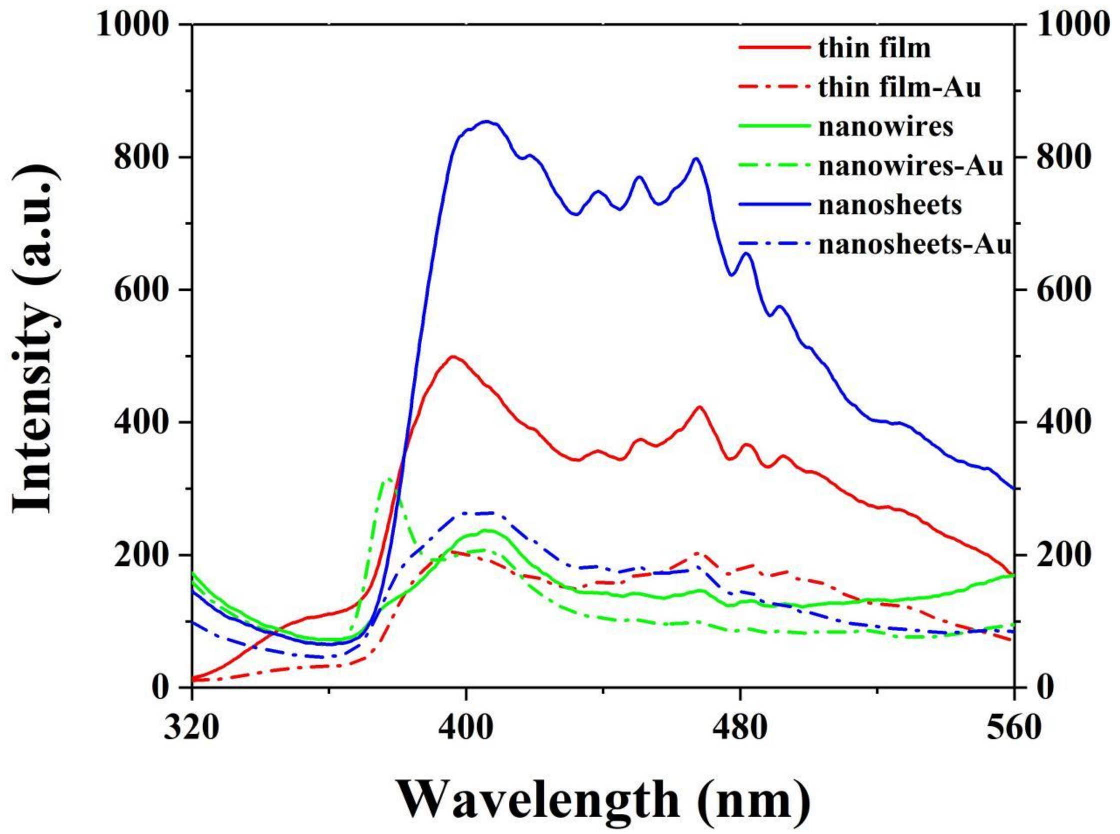

Figure S1. PL spectra of the ZnO nanostructures with and without Au NPs.

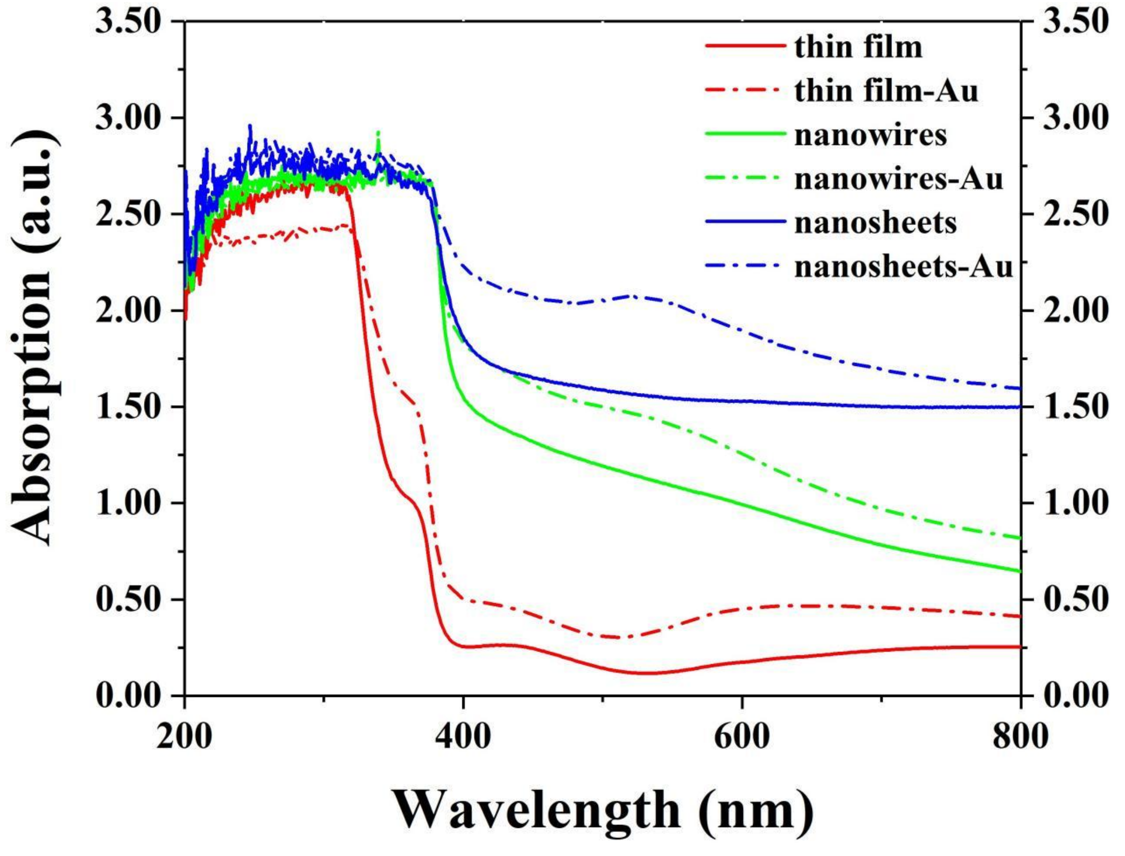

Figure S2. Absorption spectra of the ZnO nanostructures with and without Au NPs.
